# Supplementary material for: Iterative improvement in the automatic modular design of robot swarms
Source: PeerJ Comput Sci. 2020 Dec 7;6:e322. doi: 10.7717/peerj-cs.322 (PMC7924708; doi:10.7717/peerj-cs.322)
Supplement: Supplemental Information 3 [file peerj-cs-06-322-s003.zip › argos3/doc/api/standalone/a00553_source.html]

ARGoS: plugins/robots/mini-quadrotor/simulator/miniquadrotor\_rotor\_default\_actuator.h Source File


- Main Page
- Related Pages
- Namespaces
- Classes
- Files

- File List
- File Members

# plugins/robots/mini-quadrotor/simulator/miniquadrotor\_rotor\_default\_actuator.h

Go to the documentation of this file.

```
00001 
00007 #ifndef MINIQUADROTOR_ROTOR_ACTUATOR_DEFAULT_H
00008 #define MINIQUADROTOR_ROTOR_ACTUATOR_DEFAULT_H
00009 
00010 #include <string>
00011 #include <map>
00012 
00013 namespace argos {
00014    class CMiniQuadrotorRotorDefaultActuator;
00015    class CRotorEquippedEntity;
00016 }
00017 
00018 #include <argos3/plugins/robots/mini-quadrotor/control_interface/ci_miniquadrotor_rotor_actuator.h>
00019 #include <argos3/core/simulator/actuator.h>
00020 #include <argos3/core/simulator/entity/composable_entity.h>
00021 #include <argos3/core/utility/math/rng.h>
00022 
00023 namespace argos {
00024 
00025    class CMiniQuadrotorRotorDefaultActuator : public CSimulatedActuator,
00026                                               public CCI_MiniQuadrotorRotorActuator {
00027 
00028    public:
00029 
00030       enum MINIQUADROTOR_ROTOR {
00031          NORTH_ROTOR = 0,
00032          WEST_ROTOR,
00033          SOUTH_ROTOR,
00034          EAST_ROTOR
00035       };
00036 
00037    public:
00038 
00039       CMiniQuadrotorRotorDefaultActuator();
00040 
00041       virtual ~CMiniQuadrotorRotorDefaultActuator() {}
00042 
00043       virtual void SetRobot(CComposableEntity& c_entity);
00044 
00045       virtual void Init(TConfigurationNode& t_tree);
00046 
00047       virtual void SetRotorVelocities(const CCI_MiniQuadrotorRotorActuator::SVelocities& s_velocities);
00048 
00049       virtual void Update();
00050 
00051       virtual void Reset();
00052 
00053    protected:
00054 
00056       virtual void AddGaussianNoise();
00057 
00058    protected:
00059 
00061       CRotorEquippedEntity* m_pcRotorEquippedEntity;
00062       
00064       CRandom::CRNG* m_pcRNG;
00065       
00067       Real m_fNoiseStdDeviation;
00068 
00069    };
00070 
00071 }
00072 
00073 #endif
```

---

Generated on 10 Jul 2018 for ARGoS by 
 1.6.1
